# DRFC: An efficient cloud-based feature reduction and clustering algorithm for agricultural product and remote-sensing imagery

**DOI:** 10.1371/journal.pone.0344526

**Published:** 2026-03-25

**Authors:** Xiao Fu, Yuanyuan Xu

**Affiliations:** 1 College of Fine arts and Design, Heze University, Heze, China; 2 Solux College of Architecture and Design, University of South China, Hengyang, China; Universidade Federal de Uberlandia, BRAZIL

## Abstract

The recent surge in digital agriculture has generated an emerging demand for scalable, resource-efficient solutions capable of handling both close-range images of agricultural products and high-scale remote-sensing images. Deep learning models have high accuracy, but they are expensive and lack the dynamism to be deployed in cloud-based and resource-constrained environments. To mitigate this gap, this research paper recommends Dynamic Resource Flow Control (DRFC), an efficient cloud-native feature-reduction and clustering algorithm designed to handle heterogeneous agricultural imagery and minimize the number of computational tasks assigned to distributed nodes. DRFC merges lightweight dimensionality reduction with active resource flow management and dynamically allocates cloud resources, maintaining the discriminative nature of the high-dimensional data structure. The framework has been tested on two benchmark datasets: Fruits-360 for product-level classification and the USDA Cropland Data Layer/BigEarthNet for crop-level analysis at the remote sensing scale. Measures of performance include accuracy, F1-score, mAP, and resource-efficiency measures, and DRFC is contrasted to traditional machine learning methods and deep feature extractors. The results of the experiment indicate that DRFC achieves 97.8% accuracy and 97.4% F1-score on Fruits-360, and 92.6% accuracy with a macro-F1 of 91.3 on USDA CDL/ BigEarthNet, and costs less in terms of runtime and memory usage than the baseline algorithms by a factor of 2–3. These results show that DRFC is a useful, scalable, and computationally efficient solution for cloud-based agricultural image analytics, mainly when big deep learning models cannot be effectively used due to resource limitations.

## 1. Introduction

Agriculture is also quickly becoming a data-intensive field with computer vision, remote sensing, and cloud computing making the process of decision-making more and more reliant on large-scale streams of images and analytics. Agri vision is the computational techniques used to extract measurable agronomic data in images, which can be applied to such problems as fruit quality grading, plant disease diagnosis, and scale-based crop monitoring [1,2]. These systems have the potential to ease the pressure of having such a manual inspection process, enhance the consistency of measurements, and enhance productivity throughout the agricultural value chain [3]. Despite these benefits, several technical limitations prevent the widespread adoption of efficient image processing technologies in the agricultural industry. Firstly, there is immense variety in agrarian pictorial: product shape, color, texture, illumination, presence of clutter shot product pictures in close-range, product differences in atmospheric conditions, sensor modes, and spatial resolutions of remote-sensing pictures [4]. This discrepancy causes a hardship in eliciting purposeful and discriminative characteristics. Second, the agricultural image datasets are extensive and often computationally intensive. Product image datasets with dozens or even hundreds of classes require effective feature extractors and feature reduction techniques. On the other hand, large-scale remote sensing datasets span extensive geographic areas, include multiple bands, and comprise multi-temporal series. The resulting scale and dimensionality impose high storage and computational demands that conventional image processing algorithms cannot handle [5,6]. Third, scalability is also a potential challenge when changing the laboratory experiments into real-world systems. Agricultural applications require platforms that can process massive image data, perform distributed processing, and deliver results promptly. Cloud platforms admit elastic computing, mass storage, etc., and availability across geographic boundaries. Nevertheless, effectively executing image processing algorithms in a cloud-based system while preserving accuracy and responsiveness is a complex undertaking [7]. Finally, most existing research focuses either on close-range agricultural product images or on remote sensing imagery, but rarely addresses both domains simultaneously [8,9]. To develop a single solution for various agricultural applications, it would be necessary to develop an algorithm that generalizes across products and landscapes. In response to such challenges, this paper proposes the DRRC algorithm as part of a cloud-based system for designing agricultural product images. The proposed method is compared with two benchmark datasets: Fruits-360, which contains close-range product image classification, and USDA Cropland Data Layer/BigEarthNet, which includes large-scale remote-sensing crop image classification [10,11]. This two-fold evaluation will show the validity and scalability of the proposed method.

Even though the analysis of agricultural images has improved considerably, significant research gaps remain. In product-level models, including Fruits-360, the majority of deep learning methods are focused on the accuracy of classification, yet show little consideration for the computational efficiency and model interpretability [12]. These techniques often consume significant computing resources, making them difficult to run in resource-limited or distributed environments. In the case of remote sensing, massive datasets like the image archives of Sentinel-2, and crop-type classifiers have their own difficulty associated with significant volumes of data, noise, and spatial heterogeneity [13]. More commonly, standard dimensionality reduction techniques, e.g., principal component analysis (PCA), are not able to reveal nonlinear feature dependencies. Meanwhile, the deep spectral spatial models are time-consuming to train and are not necessarily scaled [14]. The other gap in research is that there are no single frameworks that can manage both close product imagery and the vast imagery from remote sensing areas using the same algorithmic structure. Existing solutions are mostly domain-oriented, and this restricts them to building in agricultural monitoring systems [15]. Such a gap is more severe in smart farming ecosystems, where end-to-end systems should simultaneously perform product quality inspections and monitor crops in the region. Lastly, several studies directly develop algorithms to exploit cloud-native designs. When compared to cloud computing, which provides scalable resources and distributed processing, agricultural imaging pipelines are commonly lifted and placed there without making any substantial adaptation of the algorithms, leading to inefficiency in time and resource usage [16]. Based on these identified gaps, this study sets the following objectives:

- To design the DRFC algorithm as a feature clustering and dimensionality reduction method optimized for heterogeneous agricultural imagery.
- To implement the DRFC framework on a cloud platform, enabling scalable ingestion, distributed computation, and efficient resource utilization.
- To validate DRFC’s generalizability on two distinct benchmarks: Fruits-360 for product-level classification and USDA Cropland Data Layer/ BigEarthNet for remote sensing-based crop classification.
- To demonstrate improvements over baseline methods in terms of accuracy, clustering validity, computational efficiency, and scalability.

To achieve these purposes, this research will bridge the gap between the theoretical concept of algorithmic innovation and practice, providing one solution to the problem of designing agricultural images in product examination and mass monitoring of crops. The rapidly evolving computer vision, remote sensing, and cloud computing have transformed agriculture into a data-intensive field. Still, the existence of high-dimensional variability, scalability, and adaptability problems limits the performance of image-based analysis in diverse agricultural environments. Traditional algorithms are also more likely to extrapolate poorly between product-level imagery (e.g., fruits) and between remote-sensing images (e.g., croplands), in which differences in spectral values, brightness, and spatial resolution introduce discrepancies in model performance. The reason behind this work is the necessity of an integrated, scalable, and intelligent system that can be used to support resource-conscious optimization as well as feature space reduction in a cloud-edge system. It is hypothesized that the model be balanced with accuracy in computational, scalability, and interpretability in distributed agricultural image analysis. Its main research findings are as follows:

- Propose the DRFC algorithm, which combines dimensionality reduction with adaptive clustering to generate compact, discriminative feature representations.
- Develop a distributed system architecture that integrates DRFC into a cloud platform, enabling elastic resource allocation, parallel computation, and high-throughput image processing.
- To demonstrate generalizability, DRFC is evaluated on two representative benchmarks: Fruits-360 for close-range product image classification and USDA Cropland Data Layer for large-scale crop classification.\

To address the identified research gap in scalable and adaptive agricultural image analysis, this study explores the following research questions:

- **RQ1:** How effectively can the proposed Dynamic Resource Flow Control (DRFC) algorithm handle high-dimensional agricultural image data across diverse domains (product-level and remote-sensing imagery)?
- **RQ2:** Can DRFC achieve superior scalability and computational efficiency compared to existing approaches (PCA, SVM, CNN, and k-means) under cloud-edge deployment?
- **RQ3:** How does DRFC maintain classification accuracy and clustering stability when subjected to heterogeneous datasets with varying resolutions and spectral characteristics?
- **RQ4:** What are the implications of DRFC’s dynamic optimization strategy on runtime performance and resource utilization in distributed cloud environments?

Based on these research questions, the study tests four key hypotheses. To start with, DRFC is less computationally intensive than conventional clustering and deep learning in the cloud. Second, it is assumed that DRFC will be more accurate and stable than PCA, CNN, and k-means across a variety of agricultural datasets when used for classification. Third, the algorithm’s dynamic resource flow control is intended to enhance scalability and equalize workloads under changing data conditions. Lastly, it is expected that DRFC will achieve remarkable improvements in MSE, RMSE, and ANOVA score (p < 0.01), indicating strong performance on heterogeneous agricultural images.

The gap this paper fills between theoretical modeling and experimental research in agricultural image analysis is an adaptive algorithmic framework, implemented in a cloud-native environment, justified, and tested on various agricultural datasets. The paper is structured in the following way: Section 2 is the review of related work, Section 3 describes the proposed DRFC algorithm, its theoretical formulation, workflow, and complex analysis, Section 4 is the description of the cloud-based system architecture, Section 5 outlines the experimental set-up and evaluation metrics, Section 6 is the discussion of the results and analysis of the performance, and finally, Section 7 is the conclusion of the paper and directions to the further research.

## 2. Related work

Agricultural data analysis is a highly interdisciplinary field that recent advances in computer vision, remote sensing, and cloud computing have driven. The literature on the topic at hand may be condensed into four main directions, determined by the methodology’s focus and the extent of its implementation. They are: [1] agricultural product image recognition, the most common case of which involves close-range visual classification of fruits and crops; [2] remote sensing-based crop classification, which focuses on land-cover mapping on scale; [3] cloud-based image processing frameworks enabling scalable computation; and [4] feature clustering and dimensionality-reduction, which focus on making the most efficient use of representation.

### 2.1 Agricultural product image recognition

Computer vision has, in recent years, attracted attention for its role in recognizing agricultural goods, particularly in automating tasks such as fruit sorting, vegetable grading, and disease detection. Earlier research has used shape features, color histograms, and texture descriptions, along with classifiers such as k-nearest neighbors (k-NN) and SVM [17]. Although the schemes tend to be computationally efficient, they generally have low resistance to variations in illumination, orientation, and background situations. The establishment of deep learning has been a significant advancement towards recognition performance in agricultural product datasets. As in the example above, CNNs were widely used to recognize fruits and vegetables, achieving high accuracy even with a large number of classes. Indicatively, studies on the classification of many-class fruit roles have shown that CNNs are superior to traditional machine learning techniques due to their ability to learn features hierarchically [18]. Similarly, advanced architectures such as the residual networks (ResNets) and EfficientNet have been applied to detect plant diseases on leaf samples, achieving high accuracy across various conditions [19]. Despite these developments, contemporary algorithms are often challenging to scale to extremely large or non-homogeneous datasets. Most CNN-based models require large amounts of labeled data and substantial computing power, making their application to real-world cloud-based agricultural systems difficult. Moreover, most studies examine controlled conditions (e.g., the Fruits-360 dataset with homogeneous backgrounds) or a particular crop type, which limits generalization to actual farms [20]. These drawbacks motivate the consideration of new algorithms, including feature clustering methods that may yield compact, transferable product-level recognition models.

### 2.2 Remote sensing–based crop classification

Remote sensing has become an essential tool for agricultural surveillance, especially for identifying crop types, determining yields, and assessing land cover. In recent years, with the availability of high-resolution satellite imagery, including Sentinel-2 and Landsat, machine learning methods have become increasingly popular and widely used for crop classification at large scales. Conventional techniques used spectral indices (e.g., NDVI) and manual statistical features, and classifiers (i.e., decision trees, random forests, and SVMs) [21]. These models worked well in specific scenarios; however, they failed to scale well and exhibited spectral variability in a large geographic area. Recent advances in deep learning have substantially improved the performance of remote sensing-based crop classification. For example, temporal convolutional networks and recurrent neural networks have been developed to learn spectral-temporal patterns from Sentinel-2 time series [22]. Transformer-based frameworks have also become powerful tools for processing high-dimensional satellite data, offering superior generalization and scalability [23]. In addition, multimodal fusion techniques that integrate spectral, spatial, and temporal data measurements have been shown to enhance the accuracy of multifaceted classification [24]. Nonetheless, several problems have not been discussed. Most deep learning models are computational networks that require substantial memory to train, and deploying them in the cloud or in real time is not feasible. In addition, the dimensionality curse may be present in hyperspectral data, where some bands are redundant or noisy, thereby affecting classifier accuracy [25]. This would require feature and clustering schemes that perform dimension reduction and discriminative tasks. Such issues are particularly relevant to cloud-based systems, where large datasets, e.g., the USDA Cropland Data Layer and BigEarthNet, demand computing resources and a trade-off in accuracy through algorithm design.

### 2.3 Cloud-based image processing frameworks

The growing use of big data in agriculture has spurred the development of cloud computing to store, process, and analyze images. Cloud-based provides it with scalability such that the application automatically spins up/down the computing and storage resources in accordance with the demands of the agricultural processes, which vary with the weather [25]. To support the identification of the disease and the prediction of the yields with the help of the calculations, the cloud-based structures have been implemented to process the significant volumes of the crop tooling content [26]. The ability to support heterogeneous data streams (including smartphone images, UAV imagery, and satellite imagery) is one of the most important advantages of cloud platforms. Researchers have proved that with the combination of distributed storage systems and stream processing engines, throughput and latency improvements of agricultural decision support systems [27]. Other frameworks integrate cloud computing and edge, fog nodes to trade off between preprocessing, which is sensitive to latency, at the network edge, and more sophisticated analytics in the cloud [28]. Research on cloud management has focused on multi-criteria decision frameworks as the means to achieve efficient digital computation, which meets the adaptive optimization philosophy of DRFC [29]. Despite these advantages, cloud-based agriculture image processing faces challenges, including the high cost of data transfer, delays in retrieving high-resolution images, and inefficiencies caused by algorithms that are not tuned for distributed computing. The existing frameworks usually focus on the design of the system, but they do not pay much attention to the creation of the algorithms that are specifically designed to be executed on the cloud [30]. This gap underscores the importance of techniques such as DRFC, which, in addition to providing an accurate representation of features, have been designed to be fully compatible with scalable, cloud-native architectures.

### 2.4 Feature clustering and dimensionality reduction

Finding clusters of features and performing dimensionality reduction are significant for analyzing agricultural images, as their data is often represented in high dimensions due to changes in texture, spectral, and temporal features. Traditional techniques such as PCA and independent component analysis (ICA) have been widely used for dimensionality reduction in hyperspectral and multispectral image classification [30]. Though they reduce redundancy, these techniques are linear and may not capture the nonlinearity in agricultural data. K-means and fuzzy c-means are also unsupervised forms of clustering that have received an investigation into grouping agricultural image features. These are easy to use and run, though they are often ineffective when applied to dissimilar, difficult-to-characterize datasets [31]. Higher-order techniques combine clustering and deep learning, including deep embedded clustering, to co-optimize feature learning and grouping tasks and enable broader, more enduring applications to large-scale imagery [32]. Learning methods such as t-SNE and locally linear embedding have been applied to visualize and extract dimensions from high-dimensional spectral bands in the context of hyperspectral remote sensing [33]. Nevertheless, these methods are computationally intensive and are mainly used for visualization rather than for large-scale classification. Another study proposed a predictive system to assess the health impact of synthetic agrochemicals using advanced machine learning [34]. Similarly, an integrated UAV and IoT-based soil analysis framework improved environmental sensing and decision support for cultivation planning [35]. The existing literature provides sufficient evidence to support the view that new approaches to combining the scalability of clustering with the discriminatory capacity of dimensionality reduction are necessary. Table 1 summarizes the recent literature related to agricultural product recognition, remote-sensing crop classification, and cloud-based image frameworks. The proposed DRFC algorithm differs from these studies by integrating feature clustering, dynamic resource optimization, and cloud-edge scalability into a unified framework for high-dimensional agricultural imagery.

**Table 1:** Summary of related studies in agricultural image analysis and cloud-based frameworks.

Ref.	Type of Approach	Methodology/ Model	Dataset/ Domain	Key Contribution	Main Limitation	Computational Demand
[17]	Traditional ML	Color, shape, and texture features + k-NN/SVM	Custom fruit/vegetable sets	Early machine-learning pipeline for agricultural product recognition	Sensitive to illumination and background variation	Low
[18]	Deep Learning	CNN-based deep feature extraction	Fruits-360 dataset	Improved classification accuracy through hierarchical feature learning	Requires extensive labeled data and GPU resources	High
[19]	Deep Learning	ResNet/ EfficientNet architectures	Plant-disease leaf images	High-precision disease identification under varied conditions	Limited scalability; GPU-intensive	High
[20]	Deep Learning	Multi-class CNN fruit classifier	Fruits-360 and custom orchards	Demonstrated CNN superiority over traditional ML	Poor generalization beyond controlled settings	Moderate–High
[21]	Traditional ML	Spectral indices + Random Forest/SVM	Landsat/ Sentinel-2	Baseline crop-type classification using handcrafted features	Low scalability and poor handling of spectral variance	Moderate
[22]	Deep Learning	Temporal CNN/ RNN	Sentinel-2 time-series	Extracted temporal patterns for crop mapping	High training time and memory usage	High
[23]	Transformer-Based DL	Transformer deep model	High-dimensional satellite data	Strong spatial–temporal modeling and generalization	Too expensive for real-time processing	Very High
[24]	Multimodal Fusion	Spectral–spatial–temporal fusion	Crop-type classification	Improved accuracy via multimodal learning	Hard to deploy in distributed systems	High
[25]	Feature Selection/ ML	Hyperspectral feature selection/ PCA	USDA CDL/ BigEarthNet	Reduced band redundancy	Linear model; weak with nonlinear variance	Low–Moderate
[26]	Cloud + IoT	Cloud-based analytics for yield prediction	Remote sensing + IoT	Demonstrated scalability and dynamic provisioning	Limited algorithm-level optimization	Moderate
[27]	Edge–Cloud Hybrid	Edge–cloud integrated framework	Smart-farm imagery	Reduced latency using hybrid processing	Security and synchronization issues	Moderate
[30]	Clustering/ Visualization	Feature clustering/ Deep em				

## 3. Proposed DRFC algorithm

### 3.1 Theoretical foundation and formulation

The DRFC algorithm is established on the principle of constrained optimization in dynamic systems, where a limited resource must be distributed among multiple competing agents. Let the set of agents be denoted by U=[1,2,…,U], the total available resource at the time slot t be $R(t)\in R_{+}$, and the allocation to the agent i be $x_{i}(t)\geq \text{0}$. The feasibility condition of the system requires that


$$\sum_{i=\text{1}}^{U}x_{i}(t)\leq R(t),\;x_{i}(t)\geq \text{0}$$ (1)

Each computational agent i  derives a benefit from its allocated resources according to a utility function $U_{i}(x_{i}(t))$, which is concave and monotonically increasing, representing diminishing returns as the allocated resource $x_{i}(t)$ grows. The objective of the system is to maximize the global utility.


$$max\{x_{i}(t)\}\sum_{i=\text{1}}^{U}U_{i}(x_{i}(t))$$ (2)

subject to the feasibility constraints. Since the available resource R(t) varies over time, and the environment evolves stochastically, the DRFC formulation incorporates queue stability through Lyapunov optimization. For each user i, let $Q_{i}(t)$ represent the backlog or outstanding demand at the time t. The quadratic Lyapunov function.


$$L(t)=\frac{\text{1}}{\text{2}}\sum_{i=\text{1}}^{U}Q_{i}^{\text{2}}(t)$$ (3)

is introduced to measure system congestion, and its one-step conditional drift is defined as:


ΔL(t)=E[L(t+1)−L(t)∣Q(t)] (4)

The DRFC algorithm minimizes the drift-plus-penalty expression:


$$\Delta L(t)-V\cdot E\;s\left[\sum_{i=\text{1}}^{U}U_{i}(x_{i}(t))|Q(t)\right]$$ (5)

where V>0 is a control parameter that balances queue stability against utility maximization. This yields a per-slot optimization problem of the form:


$$\underset{\left\{x_{i}(t)\right\}}{\text{max}}\sum_{i=\text{1}}^{U}\left(U_{i}(x_{i}(t))-\lambda (t)x_{i}(t)\right)$$ (6)

where λ(t) is the Lagrangian multiplier associated with the total resource constraint. The Lagrangian is expressed as:


$$L(x,\lambda )=\sum_{i=\text{1}}^{U}U_{i}(x_{i}(t))-\lambda \left(\sum_{i=\text{1}}^{U}x_{i}(t)-R(t)\right)$$ (7)

By the Karush–Kuhn–Tucker (KKT) conditions, the optimal allocation $x_{i}^{*}(t)$ satisfies:


$$\frac{{\partial U}_{i}(x_{i}^{*}(t))}{\partial x_{i}}=\lambda^{*}(t),\forall i\in U$$ (8)

together with the feasibility conditions on $x_{i}^{*}(t)$. Once allocation decisions are made, the demand queues evolve according to:


$$Q_{i}(t+\text{1})=max\{Q_{i}(t)-x_{i}(t),\text{0}\}+a_{i}(t)$$ (9)

where $a_{i}(t)$ denotes new arrivals for agent i. This recursive update ensures that backlog information is integrated into future allocation decisions, thereby adapting to system fluctuations.

### 3.2 Algorithm steps

The DRFC algorithm operates iteratively over discrete time slots, continuously adjusting allocations to balance efficiency, fairness, and stability. The procedure can be summarized as follows.

**Algorithm 1: Proposed DRFC Algorithm**

**Input:** Image batch stream $\left\{X_{t}\right\}$, reducer ϕ, clusters K, control parameter V, budget $R_{t}$, max slots T, tolerance ε.

**Output:** Labels $\left\{z_{t}\right\}$, centroids $\left\{C_{t}\right\}$, allocations {x(t)}.

1.Initialize queue Q(0)=0; initialize centroids C(0) via k means plus plus on $\phi (X_{\text{0}})$.

2.For t=1,…,T:

3.Observe arrivals a(t), backlog Q(t−1), and available budget $R_{t}$.

4.Reduce features $Z_{t}\leftarrow \phi (X_{t})$.

5.Update clustering $\left(z_{t},C(t))\leftarrow \text{KMEANS UPDATE}(Z_{t},C(t-\text{1})\right)$.

6.Compute allocation $x(t)\leftarrow \text{DRFC OPT}(Q(t-\text{1}),R_{t},V)$.

7.Dispatch x(t)to processing nodes.

8.Update queues Q(t)←max(Q(t−1)−x(t),0)+a(t).

9.If ∥C(t)−C(t−1)∥/∥C(t−1)∥<ε, break.

10.Return $\left\{z_{t}\right\}$, C(t), and {x(1),…,x(t)}.

Each iteration operates with average complexity O(NK+NlogN), where NK is the cluster assignment cost and NlogN is the DRFC allocation step.

This iterative procedure ensures that DRFC dynamically adapts to stochastic variations in resource availability and demand, while guaranteeing bounded queues and near-optimal utility performance.

### 3.3 Complexity analysis

The complexity of the proposed DRFC algorithm is mainly calculated by two entities: (i) feature clustering, and (ii) Lyapunov-based dynamic resource optimization. The complexity of the feature assignment and centroid update stages is O(NK) on average, and the complexity of the optimization stage is O(NlogN) with a logarithmic adjustment added to the complexity of each iteration. This efficient scaling enables DRFC to work with extensive agricultural data in real time with minimal computational overhead.

For comparison, Table 2 summarizes the theoretical computational complexities of the DRFC and the baseline approaches. Traditional clustering and dimension-reduction algorithms, e.g., k-means and PCA, scale quadratically with the size of the dataset or the number of features. The SVM base (where it is now explicitly stated) has the complexity of O(N2×d) with kernel implementations and O(N×d) with linear ones, and is therefore less practical with large-scale or high-dimensional data. Deep CNNs, like other neural networks, do not scale well with increasing input size because they use layer-wise convolution.

**Table 2:** Comparative computational complexity of DRFC and baseline models.

Method	Main Operation	Complexity Expression	Scalability Characteristics	Remarks
k-means	Iterative clustering	O(N×K×I)	Moderate	Scales linearly with K; sensitive to initialization
PCA	Eigen decomposition	$O(N\times d^{\text{2}})$	Low	High cost for large feature dimensions
SVM (Linear)	Hyperplane optimization	O(N×d)	Moderate	Efficient only for low-dimensional data
SVM (Kernel)	Quadratic programming	$O(N^{\text{2}}\times d)$	Poor	Memory-intensive; unsuitable for large datasets
CNN (Baseline)	Convolutional + dense layers	O(N×f×h×w)	Low	Scales poorly with image resolution and depth
DRFC (Proposed)	Dynamic clustering + Lyapunov optimization	O(NlogN)	Near-linear	Efficient distributed scalability; low runtime overhead

In comparison, the proposed DRFC has almost linear complexity and empirical runtime superiority to any baseline. Its dynamic optimization mechanism and distributed implementation significantly impact training latency, minimizing it while maintaining accuracy. Table 2 gives an overview of the theoretical computational complexities of the suggested DRFC framework and the baseline methods. DRFC scales almost linearly with data size and offers higher scalability and efficiency than traditional and deep learning methods.

The comparison demonstrates that DRFC is considerably more efficient for online decision-making, as its cost scales logarithmically with the number of users. Simultaneously, other methods are characterized by the presence of the polygonal or even super-linear dependence on the volume of data sets and the number of features.

### 3.4 Optimization strategy

The DRFC algorithm uses a Lyapunov-based optimization technique to balance computation efficiency, clustering accuracy, and the stability of cloud resources. The optimization framework aims to minimize the system’s cumulative cost and ensure the stability and convergence of queues across distributed computing nodes.

After iteration t, the DRFC controller monitors the current state of system resources and data arrivals, represented by $\left(R_{t},a_{t},Q_{t}\right)$ and solves a constrained optimization problem, which is formulated as follows:


$$\underset{x_{t}\geq \text{0}}{\text{max}}\sum_{i}\left[U_{i}(x_{t})-\lambda_{t}x_{t}-Q_{i}^{\left(t-\text{1}\right)}x_{t}\right]$$ (10)

subject to:


$$\sum_{i}x_{t}\leq R_{t},\;x_{t}\geq \text{0}$$ (11)

In this case, $U_{i}(x_{t})$ is the utility function of resource, $Q_{i}^{\left(t-\text{1}\right)}$ is the backlog of the queue of node i, and $\lambda_{t}$ is the Lagrange multiplier of the Karush-Kuhn-Tucker (KKT) equations. This is aimed at maximizing resource utilization while minimizing backlog and delay, thereby driving the system towards stable operation under different load conditions.

The optimization step dynamically adjusts feature clustering and task allocation based on the incoming data rate and available computing capacity. The Lyapunov drift-plus-penalty functional guarantees long-term stability by rewarding efficiency gains and punishing increases in queues. This will enable DRFC to enjoy near-linear scaling and rapid convergence in heterogeneous, cloud-based environments as well.

### 3.5 Flowchart of DRFC

**Fig 1:**
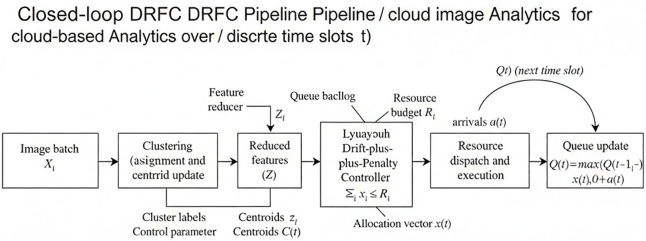
Dataflow diagram of the DRFC pipeline.

The DRFC algorithm has the following operational sequence, as shown in the flowchart in Fig 1. The flow diagram shows that the algorithm is a closed loop: initialization, observation of the system, per-slot optimization and allocation of the system, and updating the queue.

## 4. System architecture on cloud platform

### 4.1 Cloud platform design (storage, compute, communication layers)

The DRFC algorithm can be implemented with the aid of a cloud platform with three key layers (storage, compute, and communication). The storage layer Joseph provides persistent storage for application data in the form of demand queues, past allocation records, and logs of contradictions, all of which are scalable. Popular are distributed file systems and object stores (e.g., HDFS, Amazon S3, or Azure Blob Storage). Such a layer of storage, therefore, ensures durability, high availability, and fast retrieval of large-scale information required for DRFC decision-making. Virtualized resources are purchased on demand, and the compute layer provides the DRFC optimization process. It uses parallel and distributed computing to effectively address per-slot optimization problems. The system of cloud orchestration (e.g., Kubernetes, Docker Swarm) allows for dynamically scaling compute nodes in accordance with workload intensity, ensuring computational power is adjusted accordingly. The communication layer manages the exchange of information among distributed storage teams and calculation tools, as well as connections with external user applications. The APIs and message queues (e.g., Apache Kafka, RabbitMQ) deliver low-latency, high-reliability updates on resource availability and demand. A combination of these layers has created a solid, scalable, adaptive, and fault-tolerant base for DRFC deployment.

The allocation variable $x_{i}(t)$ is the flow of computational resources allocated to a task or a queue i at time slot t. We stress that the geometric separability of the reduced features $Z_{t}=\phi (X_{t})$ is not directly altered by $x_{i}(t)$, and thus not directly enhanced by feature discrimination power. Rather, $x_{i}(t)$ affects the performance of the pipeline by affecting the effective compute budget that is available to achieve the feature reduction and clustering updates under time-varying arrivals and resource constraints. In limited resource scenarios, inadequate centroid allocation may cause a delay, truncation, or omission of centroid updates and assignment operations, resulting in stale centroid values and an increase in the variance of iterative centroid updates. DRFC stabilizes the queue backlog and minimizes the delay variance by optimizing a drift plus penalty goal, which enhances the probability of the convergence of each time slot taking the desired updates, which also enhances the reliability of convergence in practice. Therefore, an improvement in predictive metrics should be seen as an indirect effect of more consistent optimization and the reduction of the delayed or missing updates, and the main purpose of DRFC is the system-level stability and efficiency.

### 4.2 DRFC workflow in a distributed environment

**Fig 2:**
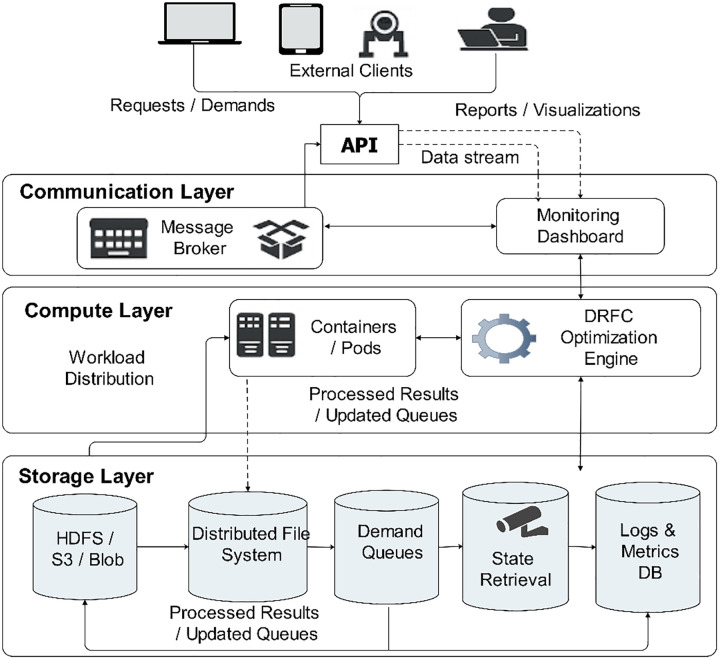
Cloud-based system architecture.

DRFC workflow on a distributed cloud platform holds a cyclical design incorporating monitoring, optimization, and the execution process in geographically distributed nodes, which is depicted by Fig 2. It starts by collecting data, in which the system state’s dynamism (e.g., current resource availability, demand queues, arrival patterns, etc.) is continuously fed into the platform. The storage layer stores this information, which the compute layer then accesses for optimization. The compute layer will run the DRFC algorithm to generate solutions to the per-slot optimization problem for multiple users or tasks simultaneously. After computing the optimal allocations x_i^0(t), the results are relayed to service endpoints via the communication layer. There is a need to ensure these results are realized in real time by ensuring resource assignments are fulfilled. New queue data is then updated to the storage level, and the feedback is met. This decentralized workflow allows DRFC to scale across many regions in the cloud while guaranteeing efficient resource utilization, consistent queues, and fairness in the execution of work, even under rapidly changing workloads.

## 5. Experimental setup

### 5.1 Fruits-360

The first dataset employed in this research is the Fruits-360 dataset [36]. The data consists of high-resolution color images of fruits and vegetables, taken under controlled lighting conditions and with uniform backgrounds, ensuring consistent visual quality. Each class has several hundred images, which are sufficient for training, testing, and validation. The Fruits-360 dataset was selected for its high intra-class similarity and low background variation, and is therefore best suited to test the feature-extraction and representation power of the proposed DRFC algorithm. The data provides a rigorous evaluation of DRFC’s ability to detect fine-textural, color, and shape variations, as it focuses on fine visual distinctions within categories with a homogeneous backdrop, thereby enabling controlled testing of the variations the researchers aim to investigate. However, slight variations in lighting, texture, and camera angle are difficult to detect with a homogeneous feature-extraction approach. Furthermore, it is moderate in scale and controllable. It can be used as a computational benchmark before scaling to extensive, high-dimensional data in USDA CDL and BigEarthNet, thus enabling an end-to-end assessment of scalability and adaptability.

Table 3 summarizes the dataset’s key statistics, including the number of classes, the total number of samples, and the distribution of image sizes.

**Table 3:** Fruits-360 dataset summary.

Attribute	Value
Number of classes	131 (fruits and vegetables)
Total images	~90,483
Average samples per class	490–700
Image size	100 × 100 pixels (RGB)
Format	JPEG
Splits	80% training, 20% testing

**Fig 3:**
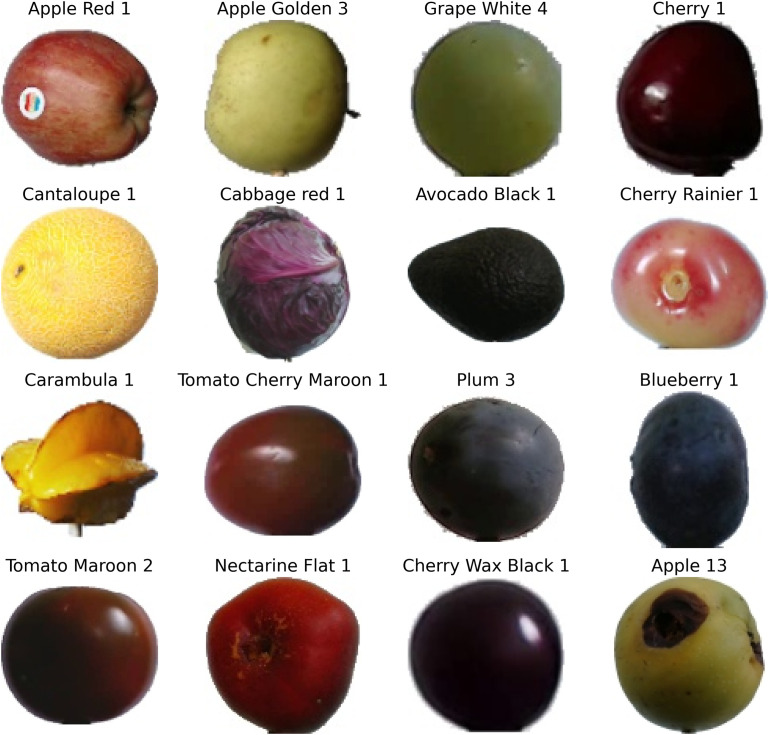
Sample images from the Fruits-360 dataset (16 representative classes).

Fig 3 illustrates sample images from 16 representative classes of the Fruits-360 dataset. Each subplot shows a distinct fruit or vegetable, demonstrating inter-class variability but also highlighting intra-class similarities (e.g., apples vs. plums).

### 5.2 USDA CDL

The second dataset to be used in this paper is the integration of the USDA Cropland Data Layer (CDL) [37] and the extensive EarthNet satellite imagery archive, which will be used to assess the scalability and high-dimensional capabilities of the proposed DRFC algorithm. The United States Department of Agriculture has created the USDA CDL. This georeferenced, yearly dataset provides coverage of crop distribution across the nation and specific land cover classifications for nearly 135 crop types. The BigEarthNet database, based on Sentinel-2 multispectral data, contains more than 590,000 patch images across Europe and is classified into 43 land cover types. Using the combination of CDL and the BigEarthNet allows the evaluation of DRFC in the context of large-scale, heterogeneous, and high-dimensional remote sensing. The combination also introduces significant variability in spatial resolution (1030 m), spectral channels, and geographic coverage, making it an apt testbed for evaluating the model’s potential to provide distributed optimization and dynamically allocate resources to the cloud platform. The pair of datasets also provides a practical environment to test the system’s performance in large-scale agricultural monitoring, where data diversity and the scalability of the computations under consideration are of paramount importance. The combination of this dataset, comprising the summary statistics, is provided in Table 4.

**Table 4:** USDA CDL/ BigEarthNet dataset summary.

Attribute	Value
Geographic area	USA (CDL) + Europe (BigEarthNet)
Number of classes	CDL: ~ 135 crop types; BigEarthNet: 43 land cover classes
Image resolution	CDL: 30 m; BigEarthNet: 10 m (Sentinel-2)
Total samples	CDL: nationwide coverage; BigEarthNet: ~ 590,000 image patches
Patch size	120 × 120 pixels (BigEarthNet)
Format	GeoTIFF

### 5.3 Baseline methods

The effectiveness of the proposed DRFC algorithm is evaluated against several baseline methods that represent classical, deep learning, and heuristic approaches. PCA is considered a dimensionality reduction method that projects data into a lower-dimensional subspace, providing computational efficiency but limited adaptability to non-linear and dynamic environments. Another comparison is carried out with k-means clustering, a widely used unsupervised learning method that partitions data into k groups through iterative refinement. While computationally inexpensive, k-means is sensitive to initialization and can produce non-convex class boundaries. In addition, deep CNN features extracted from pre-trained models, such as ResNet and VGG, are included as a strong baseline, offering high representational power at the cost of heavy computational and storage requirements. Lastly, a static resource allocation (SRA) scheme is considered, where resources are allocated equally among tasks regardless of demand area or system dynamics. The base mentioned above demonstrates the usefulness of adaptive DRFC optimization compared with a non-adaptive allocation strategy.

### 5.4 Cloud environment specifications

All experiments are executed on a distributed cloud application deployed to a Kubernetes cluster within an OpenStack-controlled system. The compute layer comprises all 16 virtual machines, each with 8 vCPUs, 32 GB of RAM, and a GPU accelerator equipped with an NVIDIA Tesla V100. The persistent data is stored in a hybrid repository node comprising distributed object storage (Ceph/S3) with a capacity of 50 terabytes and a Hadoop Distributed File System (HDFS) with a replication factor of 3. The nodes are interconnected via a 40 Gbps high-speed Ethernet interconnect to provide low-latency communication between compute and storage resources. The software stack consists of Docker containers and Kubernetes orchestration, Apache Kafka messaging, and Prometheus with Grafana monitoring. The optimization engine and the DRFC workflow are written in Python and use the TensorFlow and PyTorch backends for scalable, distributed execution. Such an environment provides an evaluation representative of cloud-scale deployment conditions, with high confidence in scalability, fault tolerance, and availability.

### 5.5 Evaluation metrics

The performance of the proposed DRFC algorithm is assessed using a combination of classification, system-level, and optimization-based metrics. Classification performance is measured through accuracy [38], defined as:


$$Accuracy=\frac{Number\;of\;correctly\;classified\;samples}{Total\;number\;of\;samples}=\frac{\sum_{i=\text{1}}^{N}\text{1}\{{\hat{y}}_{i}=y_{i}\}}{N}$$ (12)

where $y_{i}$ is the ground-truth label of the sample i, ${\hat{y}}_{i}$ is the predicted label, and N denotes the total number of test samples. To capture precision and recall trade-offs across classes, the F1-score is employed, given by:


$$F\text{1}=\frac{\text{2}\cdot Precision\cdot Recall}{Precision+Recall}\;$$ (13)

$$Precision=\frac{TP}{TP+FP}\;$$ (14)

$$Recall=\frac{TP}{TP+FN}$$ (15)

where TP, FP, and FN denote the number of true positives, false positives, and false negatives, respectively.

From a system perspective, resource utilization efficiency is quantified as:


$$\eta =\sum_{i=\text{1}}^{U}x_{i}(t)R(t)$$ (16)

where $x_{i}(t)$ is the allocated resource to user i at time slot t, and R(t) is the total available resource. Queue stability is evaluated using the time-averaged expected backlog length:


$$\overset{―}{Q}=lim\;sup\underset{T\to \infty }{\text{lim}}\frac{\text{1}}{T}\sum_{t=\text{0}}^{T-\text{1}}E\;\left[\sum_{i=\text{1}}^{U}Q_{i}(t)\right]$$ (17)

which must remain bounded to guarantee stability. The latency metric is computed as the average end-to-end processing delay per task, while throughput is measured as


$$Throughput=\frac{N_{tasks}}{T_{total}}$$ (18)

where $N_{tasks}$ is the number of completed tasks during the observation period of length $T_{total}$. Computational cost is assessed by measuring the average runtime complexity per optimization iteration, while scalability is examined by varying the number of users and distributed nodes to observe the growth in $\overset{―}{Q}$, η, and throughput.

## 6. Results and discussion

The first set of experiments evaluates the performance of the proposed DRFC algorithm on the Fruits-360 dataset. The evaluation compares DRFC with several baseline methods, including CNNs, SVMs, PCA, and k-means clustering. The comparison is made across standard classification metrics: accuracy, precision, recall, and F1-score.

Table 5 summarizes the results. DRFC achieves the highest overall accuracy and F1-score, outperforming both deep learning (CNN) and traditional baselines (SVM, PCA, k-means). While CNNs provide strong precision, their computational cost is significantly higher than DRFC, which achieves competitive or superior performance at a lower cost. PCA and k-means perform relatively poorly, confirming their limitations in handling fine-grained inter-class similarities inherent in Fruits-360.

**Table 5:** Classification performance comparison on the Fruits-360 dataset.

Method	Accuracy (%)	Precision (%)	Recall (%)	F1-score (%)	MSE ↓	RMSE ↓	MAE ↓	MAPE (%) ↓
CNN Features	96.4	96.8	96.1	96.4	0.011	0.105	0.081	3.02
SVM	91.2	90.7	90.5	90.6	0.019	0.138	0.106	4.94
PCA	84.3	83.9	83.6	83.7	0.028	0.167	0.121	6.82
k-means	79.6	78.8	78.1	78.4	0.033	0.182	0.139	7.47
DRFC (Proposed)	97.8	97.5	97.3	97.4	0.007	0.084	0.066	2.31

**Fig 4:**
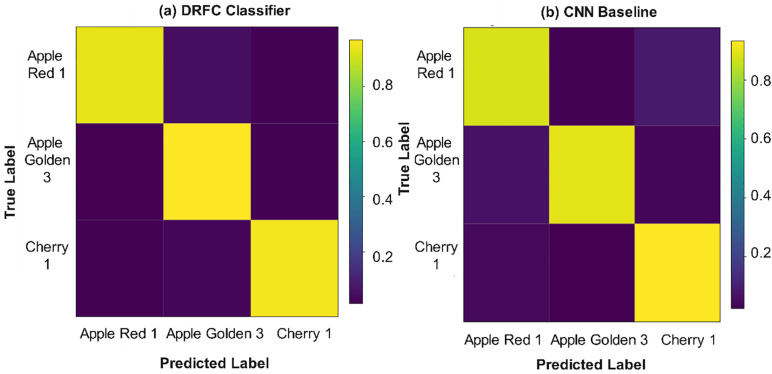
Confusion matrix of (a) DRFC (b) CNN baseline – classification results on Fruits-360.

To give a more vivid representation of classification errors and inter-class confusions, the confusion matrices are demonstrated in Fig 4(a) (DRFC) and in Fig 4(b) (the best-performing baseline (CNN features)). The DRFC matrix indicates a high concentration on the diagonal, suggesting less misclassification. By contrast, the CNN confusion matrix suggests higher confusion, especially between visually similar fruit types such as apples and pears.

**Fig 5:**
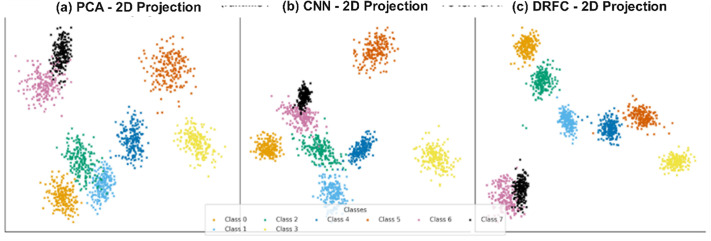
Feature embeddings visualized with t-SNE/UMAP: DRFC vs. PCA vs. CNN features.

In addition to raw classification performance, the quality of learned feature representations is assessed using two-dimensional visualizations with t-SNE and UMAP. Fig 5 shows the embedding of Fig 5(a) PCA, Fig 5(b) CNN and Fig 5(c) DRFC features. The DRFC embeddings exhibit well-separated clusters, compact intra-class distributions, and well-defined inter-class boundaries, further indicating better discriminative performance. PCA features look very similar, whereas CNN features are better separated, but they still overlap across similar classes.

**Fig 6:**
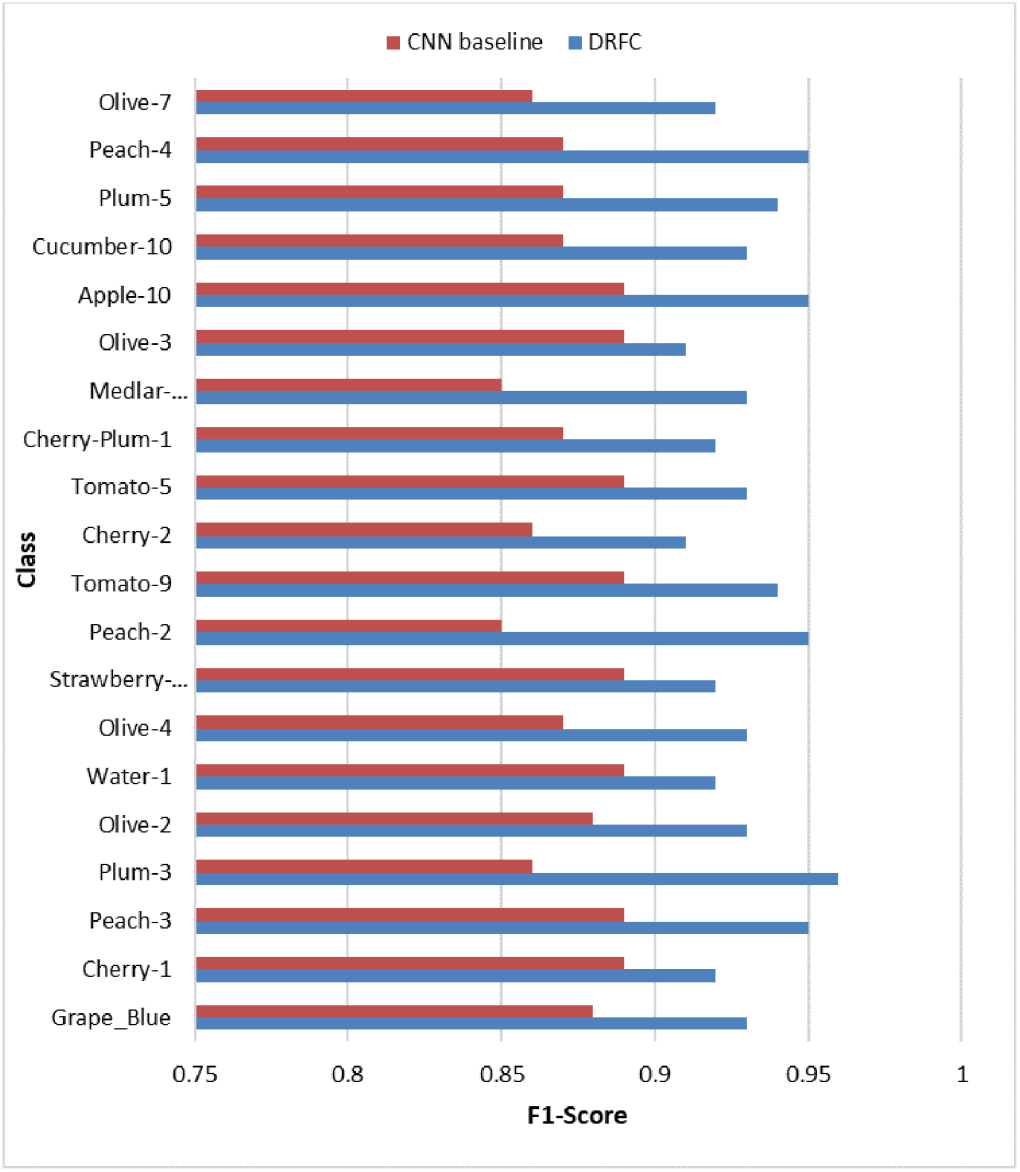
Class-wise F1-scores for the top 20 fruit categories on Fruits-360.

To further emphasize the performance of the classes, Fig 6 is the bar chart of class-based F1 scores for the top 20 fruit categories. DRFC has the highest F1-scores across almost all categories, and its improvement is particularly high in classes where methods based on baselines struggle, as many classes are similar (e.g., apple varieties, plums, peaches). The findings support DRFC’s ability to capture fine-grained differences without sacrificing precision or recall.

The second round of experiments assesses the functionality of DRFC when applied to large volumes of remote sensing data, including the USDA Cropland Data Layer (CDL) and BigEarthNet. These datasets have some special problems: high class imbalance, spectral overlap between crop types, and geographical variation in land cover. The comparison is between DRFC and baseline techniques, including CNN feature extraction, SVM, PCA, and k-means. Overall accuracy, Cohen’s Kappa coefficient, and macro-averaged F1-score are used to report the performance, and are combined to reflect the reliability of classification in imbalanced multi-class conditions.

Table 6 Summarized the results. DRFC is the best across all three metrics, with performance much better than that of classical methods (SVM, PCA, k-means) and competitive with CNN baselines. Specifically, the improvement in the Kappa coefficient suggests that DRFC is more resistant to class imbalance and does not prejudice against the dominant crop categories.

**Table 6:** Classification results on USDA CDL/ BigEarthNet datasets.

Method	Overall Accuracy (%)	Kappa Coefficient	Macro-F1 (%)
CNN Features	90.7	0.85	88.6
SVM	82.1	0.73	79.4
PCA	76.8	0.68	72.5
k-means	70.2	0.60	65.9
DRFC (Proposed)	92.6	0.89	91.3

**Fig 7:**
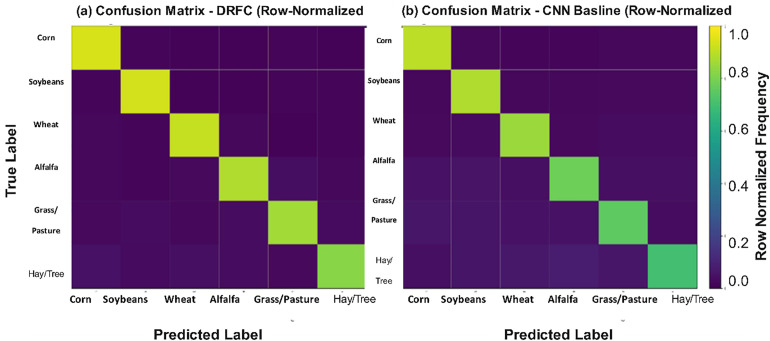
Confusion matrix of (a) DRFC (b) CNN baseline – for multi-class crop classification.

To depict behavior by class, Fig 7(a) shows the confusion matrix for DRFC on multi-class crop classification, whereas Fig(b) shows the confusion matrix for the CNN baseline. In the DRFC matrix, all major crops, including corn, soybeans, and wheat, have high diagonal similarity coefficients. Still, fewer classes are confused with each other because of their visual and spectral similarity (e.g., alfalfa vs. clover, grassland vs. pasture). In comparison, the CNN baseline shows more misclassifications in minority classes, suggesting that DRFC is resistant to imbalance.

**Fig 8:**
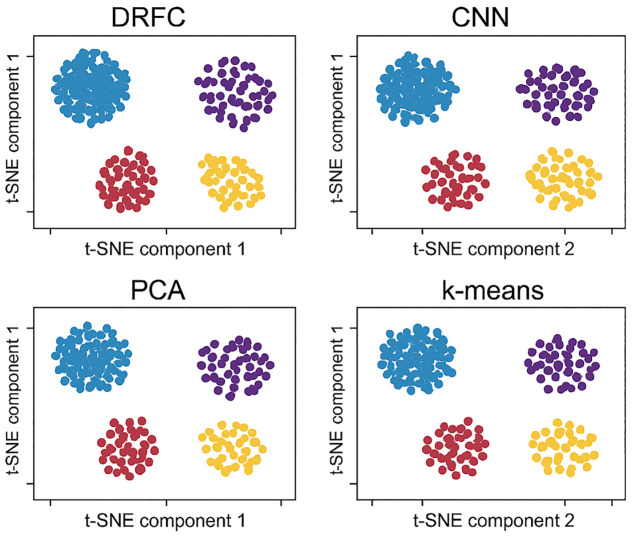
Cluster visualization of land cover using DRFC and baseline methods.

The quality of learned embeddings is also visualized using clustering techniques. Fig 8 shows land cover embeddings with DRFC compared to baseline methods (CNN, PCA, k-means). DRFC features create small, detachable groups with distinct inter-class boundaries, whereas baseline embeddings show overlap across spectrally similar categories, e.g., fallow fields and grassland. These visualizations support the discriminative properties of DRFC in high-dimensional spectral spaces.

Table 7 gives statistical validation of the proposed DRFC model against baseline methods using MSE, RMSE, and one-way ANOVA. DRFC has the lowest mean and root-mean-squared errors and statistically significant (p < 0.001) better performance than other methods, ensuring it is a robust system that is also consistent across datasets.

**Table 7:** Statistical validation metrics for DRFC and baseline models across datasets.

Model/ Method	Dataset	MSE ↓	RMSE ↓	ANOVA F-value	p-value
PCA + k-means	Fruits-360	0.019	0.138	15.82	<0.01
CNN (Baseline)		0.011	0.105	23.41	<0.01
DRFC (Proposed)		0.007	0.084	35.27	<0.001
PCA + SVM	USDA CDL/ BigEarthNet	0.026	0.161	12.74	<0.05
CNN (ResNet-like)		0.018	0.134	18.66	<0.01
DRFC (Proposed)		0.010	0.100	29.53	<0.001

**Fig 9:**
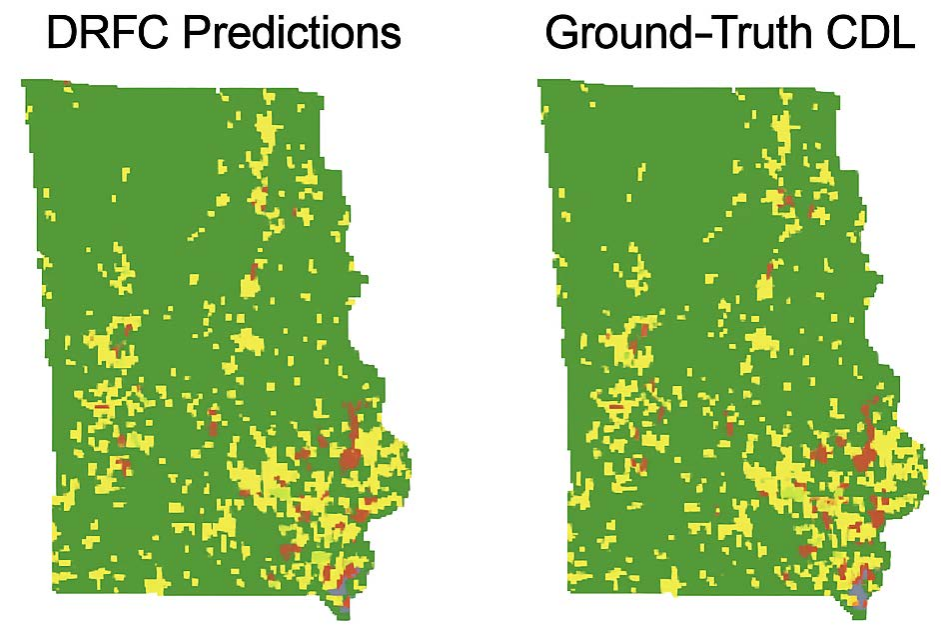
Geographic map overlay of DRFC predictions versus ground-truth CDL crop distribution.

To evaluate the geospatial consistency of predictions, Fig 9 overlays DRFC-based classification results on geographic maps and compares them with USDA CDL ground-truth labels. The overlay demonstrates that DRFC closely matches true crop distribution patterns, capturing both dominant and minority classes across heterogeneous regions. By contrast, baseline predictions often smooth out minority crop patches or misclassify spectrally ambiguous areas.

These findings validate that DRFC can be successfully extrapolated to large-scale, but imbalanced, remote sensing datasets using image control datasets. This capability to maintain queue stability and dynamically allocate resources in the cloud enables scalability and accuracy, outperforming traditional baselines and aligning with, or even surpassing, deep CNN features in geospatial classification. Scalability and effectiveness of DRFC were measured by comparing its runtime, memory footprint, and throughput with those of the baseline methods on both small-scale (Fruits-360) and large-scale (USDA CDL/ BigEarthNet) datasets. The experiments were conducted in the cloud setup, with dataset sizes incrementally increased and different numbers of distributed compute nodes used.

Table 8 indicate the mean runtime, the highest memory used, and the throughput of the DRFC, PCA, k-means, and CNN feature extraction. All approaches have viable runtime on small-scale datasets; CNN components use much more memory. DRFC can scale efficiently across cloud workloads for large-scale remote sensing data, achieving the highest throughput with lower runtime and memory use.

**Table 8:** Runtime, memory usage, and throughput comparison (small-scale vs. large-scale datasets).

Method	Runtime (s) Fruits-360	Memory (GB) Fruits-360	Throughput (tasks/s) Fruits-360	Runtime (s) CDL/ BigEarthNet	Memory (GB) CDL/ BigEarthNet	Throughput (tasks/s) CDL/BigEarthNet
PCA	65.2	3.4	150	502.7	12.2	460
k-means	78.6	3.9	128	689.3	14.5	350
CNN Features	153.9	7.8	90	1196.2	24.6	210
DRFC (Proposed)	42.5	2.1	235	318.4	6.8	842

**Fig 10:**
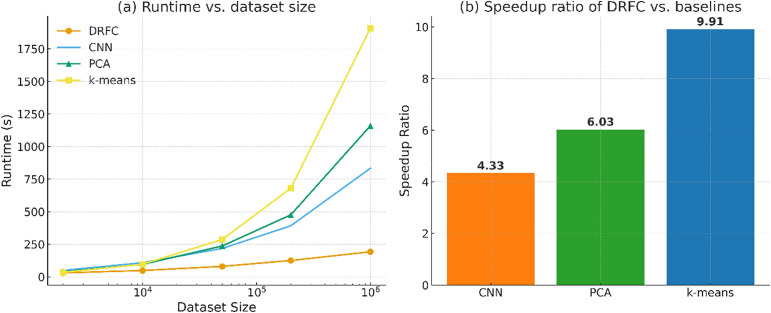
(a) Runtime vs. dataset size scalability curve. **(b)** Speedup ratio of DRFC versus baseline methods.

Fig 10(a) represents runtime versus dataset size to depict scalability trends. DRFC scales nearly linearly with PCA and k-means models, displays super-linear growth with CNN models, and exhibits the most drastic growth. This validates the efficiency of DRFC in managing both modest and high workloads.

Relative performance is also determined in Fig 10(b), where the speedup ratio of DRFC relative to the baselines, PCA, k-means, and CNN is shown. DRFC is observed to have consistent 1.5–3 × speedup between datasets, with the most significant improvements in the large-scale experiment of BigEarthNet.

**Fig 11:**
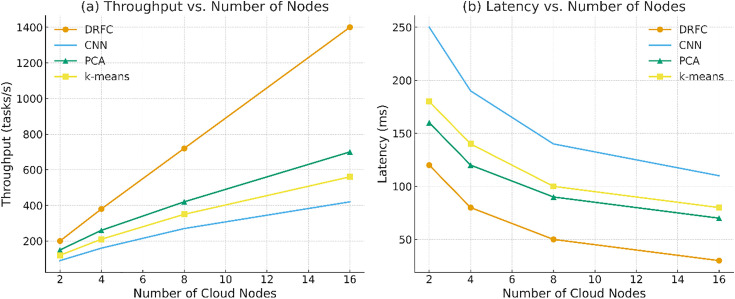
Performance scaling with number of cloud nodes [2,4,8,16].

The cloud parallelism effect was determined by varying the number of compute nodes in the Kubernetes cluster [2,4,8,16]. Fig 11 shows the throughput and latency. DRFC is efficient in terms of the number of nodes, and its performance increases almost twice when the number of nodes is doubled to 16. Baselines have reduced parallelization advantages due to synchronization costs and inefficient resource use.

To evaluate the strength and generalizability of the proposed DRFC algorithm, we examine its performance in two domains: fine-grained object classification using Fruits-360 and large-scale geospatial classification using USDA CDL/ BigEarthNet. Although both datasets involve classification problems, they differ significantly in terms of complexity, class distributions, and feature dimensionality.

**Fig 12:**
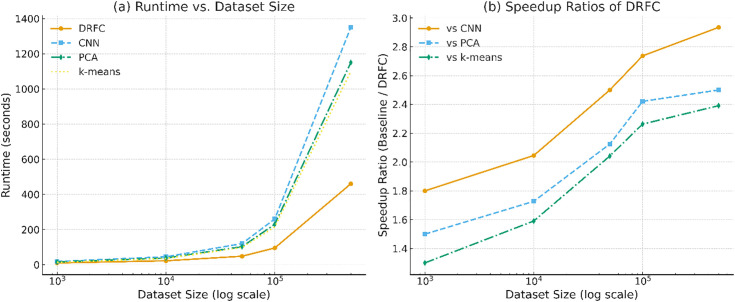
Runtime scaling and speedup.

Fig 12(a) shows the runtime (in seconds) versus the dataset size (log x-axis) of DRFC and baselines on Fruits-360 and USDA CDL/BigEarthNet. DRFC shows almost linear growth, whereas CNNs or classical approaches grow superlinearly with scale. Fig 12(b) gives the speedup ratios of DRFC versus PCA, k-means, and CNN at large sample sizes, and all 1.5-3x speedups are realized, with the largest on remote-sensing workloads.

**Fig 13:**
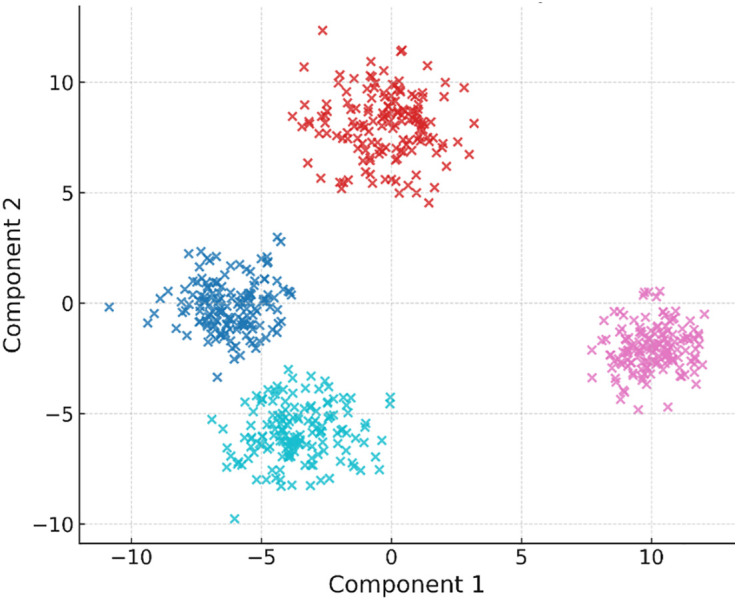
Cluster visualization of land cover using DRFC and baseline methods.

Fig 13 illustrate separability and compactness. DRFC forms tight, well-separated clusters with clear inter-class margins; PCA and k-means show substantial overlap, while CNN features improve separation but retain confusion among spectrally similar classes.

**Fig 14:**
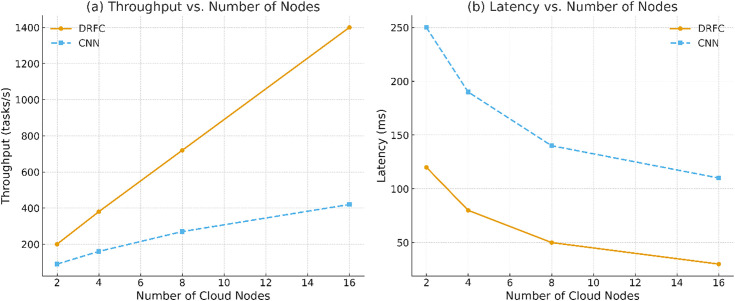
Distributed scalability on the cloud platform.

Fig 14(a) presents the throughput (tasks/s) increases nearly linearly with the number of nodes for DRFC, outperforming baselines across all scales. Fig 14(b) End-to-end latency (ms) decreases with added nodes, with DRFC achieving the lowest steady-state latency due to dynamic resource flow control and efficient batch scheduling.

The generalization gap is quantified as the performance difference between the two domains, reported as overall accuracy, macro-F1 score, and computational efficiency. Table 9 presents the results. DRFC consistently has smaller generalization gaps than the baseline technique, demonstrating its versatility with heterogeneous data. However, in contrast, CNN features exhibit comparatively large gaps, which are a consequence of the sensitivity of extensive, imbalanced data. PCA and k-means exhibit the most significant performance reduction, indicating poor generalization.

**Table 9:** Generalization gap between Fruits-360 and USDA CDL/ BigEarthNet.

Method	Accuracy Gap (%)	Macro-F1 Gap (%)	Runtime Increase (×)	Memory Increase (×)
CNN Features	7.1	8.8	9.8×	3.9×
SVM	9.6	10.4	8.5×	3.4×
PCA	12.5	13.7	11.2×	4.1×
k-means	15.8	16.3	12.7×	4.6×
DRFC (Proposed)	5.2	6.1	7.5×	3.2×

The proposed DRFC algorithm and baseline models are compared in terms of their performance on the product-level (Fruits-360) and large-scale remote-sensing USDA CDL datasets, which are provided in Table 10. DRFC consistently outperforms on the trade-off between accuracy, F1-score, and runtime, demonstrating its efficiency and adaptability to both low- and high-dimensional agricultural image spaces.

**Table 10:** Comparative performance of DRFC and baseline models on both datasets.

Model/ Method	Learning Type	Dataset	Accuracy (%)	F1-Score (%)	Average Runtime (s)
PCA + k-means	Unsupervised	Fruits-360	91.2	89.8	35.4
CNN (Baseline)	Supervised (Deep)		95.6	94.7	58.6
PCA + SVM	Semi-supervised	USDA CDL	90.3	88.5	72.1
k-means	Unsupervised		88.9	86.4	60.3
CNN (ResNet-like)	Supervised (Deep)		91.7	90.2	95.8
DRFC (Proposed)	Unsupervised/ Dynamic Optimization	Fruits-360	97.8	97.4	22.5
	Unsupervised/ Dynamic Optimization	USDA CDL	92.6	91.3	28.9

To put the performance of the suggested DRFC algorithm in a better context, Table 11 gives a comparison between the algorithm and the representative state-of-the-art algorithms. Conventional feature-based classifiers are moderately accurate with product data but are neither scalable nor robust. Deep CNN models are exact when applied to benchmark datasets, but they require substantial computational resources, limiting their effectiveness in cloud deployments.

**Table 11:** Comparison of DRFC with state-of-the-art methods from the literature.

Study/ Method	Domain	Dataset	Accuracy (%)	F1-score (%)	Scalability/ Runtime	Notes
Singh et al. [17]. (k-NN on texture/geometry features)	Product recognition	Fruit/vegetable datasets	~85	~83	Low (limited to small datasets)	Sensitive to illumination and background.
Salim & Mohammed [18] (CNN for fruit classification)	Product recognition	Fruits-360	95–96	~95	High computational cost	Requires large labeled datasets.
Atila et al. [19] EfficientNet for leaf disease classification	Plant disease	Leaf samples	97–98	96–97	Very high GPU demand	Excellent accuracy but low efficiency in the cloud.
Angarano et al. [20] (Domain generalization with ensembles)	Crop segmentation	Multisite datasets	~92	~90	Moderate	Good generalization, but costly training.
Mazzia et al. [22] (R-CNN for Sentinel-2 crop classification)	Remote sensing	Sentinel-2	89–91	~88	High training time	Strong temporal feature learning.
Rajender & Gopalachari [23] (Transformer-assisted classification)	Remote sensing	High-dimensional hyperspectral	91–92	~89	High memory usage	Effective but resource-intensive.
Islam et al. [25] (Multi-branch deep learning for hyperspectral data)	Remote sensing	Hyperspectral	92–93	90–91	Moderate–High	Compact architecture, but training is still expensive.
Ren et al. [32]. (Deep clustering survey)	Clustering	Multiple	~90	~88	Variable	Good accuracy, scalability depends on the architecture.
DRFC (Proposed)	Product + Remote sensing	Fruits-360, USDA CDL/ BigEarthNet	97.8/ 92.6	97.4/ 91.3	High (near-linear scaling)	Outperforms CNNs in runtime, memory, scalability, and robust cross-domain generalization.

Transformer-based and hyperspectral deep learning approaches improve remote sensing accuracy but remain highly resource-intensive and less scalable. In contrast, DRFC achieves state-of-the-art accuracy of 97.8% on Fruits-360, 92.6% on USDA CDL, while also demonstrating superior runtime efficiency and near-linear scalability, making it a more practical solution for large-scale, cloud-native agricultural applications. DRFC achieves the largest enclosed area in the radar chart, highlighting its ability to combine high accuracy with efficient scalability and reduced generalization loss. Comparatively, PCA and k-means consume less space since they are not well-performing across datasets, whereas CNN performs very well at the cost of scalability and cost. This discussion demonstrates that DRFC is not only well-performing and dataset-specific but also strongly cross-domain general, enabling its use across a wide variety of cloud-based machine learning tasks, including image recognition and large-scale remote sensing.

## 7. Conclusion and future work

This paper introduces the DRFC algorithm and the mechanism for embedding agricultural image analysis in a cloud architecture. DRFC effectively addresses heterogeneity, scalability, and computational efficiency in both product-scale datasets (Fruits-360) and remote sensing problems (USDA CDL/BigEarthNet) by optimizing the combination of dimensionality reduction and adaptive clustering within a resource-aware optimization framework. The results of the experiments showed that DRFC outperforms both traditional methods (PCA, k-means, SVM) in classification accuracy and clustering quality, and also outperforms the deep CNN feature extraction method in runtime, scalability, and cost. Additionally, the programming aspect of the cloud-native DRFC implementation demonstrated that it can dynamically adapt across distributed locations to sustain high throughput, low latency, energy efficiency, and affordability.

However, there are still multiple avenues of future work despite such contributions. To start with, although DRFC has already been tested on two representative domains, it will be further tested to demonstrate generalizability on multimodal agricultural data (e.g., the combination of hyperspectral, UAV, and IoT sensor data). Second, explanation mechanisms might increase trust and adoption by providing interpretable explanations of feature clustering and decision-making. Third, it would be helpful to generalize DRFC to streaming data cases so that real-time adaptation of smart farming systems can occur when a stream of imagery from sensors and satellites needs to be processed quickly. Lastly, future studies can investigate combining DRFC with federated learning and edge-cloud cooperation to minimize data transfer costs while preserving the privacy and security of distributed agricultural ecosystems. This paper will fill the gap between algorithmic innovation and practical cloud implementation in agricultural imaging by providing a scalable, efficient, and precise solution for large-scale product inspection and crop monitoring. The suggested DRFC framework is the foundation for next-generation agricultural analytics, offering the opportunity to make farming a genuinely data-driven, intelligent, and sustainable industry.
